# Effect of Anti-Obesity Drug on Cardiovascular Risk Factors: A Systematic Review and Meta-Analysis of Randomized Controlled Trials

**DOI:** 10.1371/journal.pone.0039062

**Published:** 2012-06-20

**Authors:** Yu-Hao Zhou, Xiu-Qiang Ma, Cheng Wu, Jian Lu, Shan-Shan Zhang, Jia Guo, Shun-Quan Wu, Xiao-Fei Ye, Jin-Fang Xu, Jia He

**Affiliations:** 1 Department of Health Statistics, Second Military Medical University, Shanghai, China; 2 Tumor Immunology and Gene Therapy Center, Eastern Hepatobiliary Surgery Hospital, Second Military Medical University, Shanghai, China; 3 Department of Ultrasonography, Eastern Hepatobiliary Surgery Hospital, Second Military Medical University, Shanghai, China; University of Colorado Denver, United States of America

## Abstract

**Background:**

Anti-obesity drugs are widely used to prevent the complications of obesity, however, the effects of anti-obesity drugs on cardiovascular risk factors are unclear at the present time. We carried out a comprehensively systematic review and meta-analysis to assess the effects of anti-obesity drugs on cardiovascular risk factors.

**Methodology and Principal Findings:**

We systematically searched Medline, EmBase, the Cochrane Central Register of Controlled Trials, reference lists of articles and proceedings of major meetings for relevant literatures. We included randomized placebo-controlled trials that reported the effects of anti-obesity drugs on cardiovascular risk factors compared to placebo. Overall, orlistat produced a reduction of 2.39 kg (95%CI-3.34 to −1.45) for weight, a reduction of 0.27 mmol/L (95%CI: −0.36 to −0.17) for total cholesterol, a reduction of 0.21 mmol/L (95%CI: −0.30 to −0.12) for LDL, a reduction of 0.12 mmol/L (95%CI: −0.20 to −0.04) for fasting glucose, 1.85 mmHg reduction (95%CI: −3.30 to −0.40) for SBP, and a reduction of 1.49 mmHg (95%CI: −2.39 to −0.58) for DBP. Sibutramine only showed effects on weight loss and triglycerides reduction with statistical significances. Rimonabant was associated with statistically significant effects on weight loss, SBP reduction and DBP reduction. No other significantly different effects were identified between anti-obesity therapy and placebo.

**Conclusion/Significance:**

We identified that anti-obesity therapy was associated with a decrease of weight regardless of the type of the drug. Orlistat and rimonabant could lead to an improvement on cardiovascular risk factors. However, Sibutramine may have a direct effect on cardiovascular risk factors.

## Introduction

Cardiovascular disease is the leading cause of premature morbidity and mortality worldwide for both men and women [1]–[4]. Obesity and overweight are escalating and raising considerable public concern because they increase the prevalence of severe cardiovascular events and other systemic diseases, causing great costs and burden to both society and families [5]–[7]. Over the past few decades, several randomized controlled trials have already indicated that reduction in weight could reduce the risk of cardiovascular outcomes and other systemic diseases. In practice, lifestyle modifications remain the primary approach for obesity therapy. However, lifestyle modifications have their own limitation on lasting weight loss, especially for adolescent obesity. In fact, more than 80% of the highly motivated patients are unable to achieve weight loss with dietary and lifestyle modifications alone. Lifestyle modifications are not sufficient impact on weight loss and attentions have been drawn to additional effective prevention therapies [7]–[12]. Anti-obesity drugs represent additional effective therapies which have clear effect on weight loss. However, the effects of anti-obesity drugs on cardiovascular risk factors remain unclear [7], [13]. Previous review [14] only provided evidence that anti-obesity drugs have a role in weight loss for obese adolescents, but did not provide clear evidence showing whether anti-obesity therapy was associated with effects on cardiovascular risk factors.

Recently, several randomized controlled trials [15], [16] investigating the use of anti-obesity therapy have been performed. Data from these recent trials are needed to be evaluated to formulate a conclusion regarding the efficacy of anti-obesity therapy. We therefore conducted a systematic review and meta-analysis of pooled data from randomized controlled trials, including the latest evidence of the association between anti-obesity therapy and the risk of cardiovascular risk factors and any possible adverse reactions.

## Methods

### Data Sources, Search Strategy and Selection Criteria

Randomized controlled trials and literature reporting trials of anti-obesity therapy in English met the eligibility criteria for our meta-analysis regardless of publication status (published, unpublished, in press or in progress), and relevant literatures were found by the following procedures:

(1) Electronic searches: We searched the electronic databases EmBase, Medline, and the Cochrane Center Register of Controlled Trials for articles to a time limit of September 20, 2010, using “rimonabant”, “sibutramine”, “orlistat”, “obesity” and “randomized controlled trial” as the search terms. All reference lists from reports on non-randomized controlled trials were searched manually for additional eligible studies.

(2) Other source: We contacted the authors to obtain any possible additional published or unpublished data and searched the proceedings of annual meetings in the Cochrane Obesity Group Specialized Register. In addition, we searched the ongoing randomized controlled trials, which had been registered as been completed but not yet been published, in the metaRegister of Controlled Trials. Medical subject headings and methods, patient population and intervention were used to identify relevant trials. This review was conducted and reported according to the PRISMA (Preferred Reporting Items for Systematic Reviews and Meta-Analysis) Statement issued in 2009 (Checklist S1) [17].

**Figure 1:**
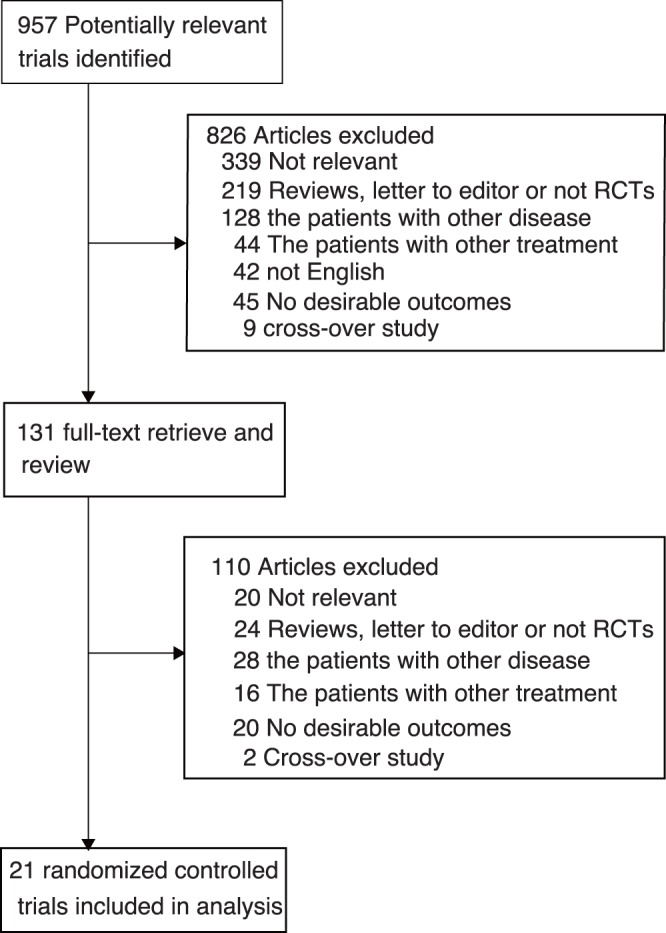
Flow diagram of the literature search and trials selection process.

**Table 1:** Design and characteristic of trials included in the systematic review and meta-analysis.

Source	No. of patients	Mean age, y	Female patients (%)	Interventions	Baseline BMI (kg/m^2^)	Duration of follow-up (month)	Jadad score
Stephen R(1998) [21]	487	NG	85.2	Orlistat 120 mg three times daily	35.0	24	3
Lars S(1998) [22]	688	44.8	83.0	Orlistat 120 mg three times daily	36.0	12	5
James O.H(1999)[23]	363	46.2	86.0	Orlistat 120 mg three times daily	32.8	12	3
Michael H.D(1999) [24]	892	43.5	84.2	Orlistat 120 mg three times daily	36.3	24	5
N Finer(2000) [25]	218	41.4	88.5	Orlistat 120 mg three times daily	36.8	12	5
W.P.T James(2000) [26]	467	40.6	83.5	Sibutramine 10 mg daily	36.6	24	4
Alfred W(2001) [27]	606	43.4	75.4	Sibutramine15 mg daily	34.8	10	6
M Krempf(2003) [28]	696	41	86.4	Orlistat 120 mg three times daily	36.1	18	4
The S.A.T Study (2003) [29]	348	42.7	74.4	Sibutramine15 mg daily	35.4	12	6
R.I Berkowitz(2003) [35]	82	14.1	55.0	Sibutramine5 or 10 mg daily	37.8	12	6
Jarl S.T(2004) [30]	3277	43.3	55.2	Orlistat 120 mg three times daily	37.3	48	4
Julie A.P(2004) [31]	588	48.3	81.6	Sibutramine10 mg daily	37.8	12	5
A Godoy-Matos (2004) [36]	60	16.2	81.7	Sibutramine10 mg daily	36.8	6	4
Chanoine J.P(2005) [32]	539	13.6	67.0	Orlistat 120 mg three times daily	35.6	12	5
RIO-Europe Study Group(2005) [37]	1507	45.0	79.5	Rimonabant 5 or 20 mg daily	36.0	12	6
Rimonabant in Obesity–Lipids Study Group(2005) [38]	1036	47.8	60.6	Rimonabant 5 or 20 mg daily	34.0	12	6
James W.A(2006) [33]	391	46.2	94.4	Orlistat 60 mg three times daily	26.8	4	5
W.S.C Poston(2006) [34]	167	40.7	91.6	Orlistat 120 mg three times daily	36.0	12	2
L.M Garcia-Morales (2006) [39]	46	15.0	56.5	Sibutramine10 mg daily	35.9	6	5
S.R Daniels (2007) [15]	498	13.7	64.7	Sibutramine 10 mg daily	36.1	12	4
ADAGIO-Lipids Investigators(2009) [16]	803	49.6	53.6	Rimonabant 20 mg daily	36.2	12	4

The literature search, data extraction, and quality assessment were undertaken independently by two authors (Xiu-Qiang Ma and Jian Lu) with a standardized approach, and any disagreement between these two authors was settled by a third author (Yu-Hao. Zhou) until a consensus was reached.

All completed randomized controlled trials assessing the effects of anti-obesity therapy with placebo as control and reporting at least one outcome of cardiovascular risk factors were included as eligible trials.

### Data Collection and Quality Assessment

One author (Yu-Hao. Zhou) designed a standard data extraction procedure, and then three authors (Cheng Wu, Jia Guo, Xiao-Fei Ye) checked each full-text trial for eligibility and extracted and tabulated all relevant data with a standardized flow path. Extracted data included patients’ baseline characteristics, the type and dose of anti-obesity drugs, follow-up duration, change in weight, cardiovascular risk factors, and any possible adverse outcomes. Disagreements regarding the data were settled by group discussion.

Study quality was assessed using the Jadad [18] scores (Jin-Fang Xu, Yu-Hao Zhou) based on randomization, concealment of the treatment allocation, blinding, completeness of follow-up, and the use of intention-to-treat analysis.

**Figure 2:**
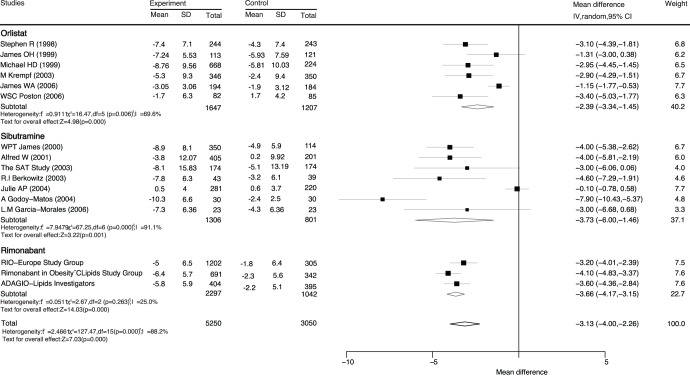
Mean reduction in weight loss (kg) based on sibutramine and orlistat. CI, confidence intervals; IV, inverse variance.

### Statistical Analysis

Relative risks (RRs) or mean differences (MDs) with 95% confidence intervals (CIs) were calculated using outcomes extracted from each trial before data pooling. We used RRs with 95% CIs for binary data to estimate the effects of anti-obesity drugs on adverse events and MDs with 95% CIs for continuous data to estimate the effects of anti-obesity drugs on weight loss, and cardiovascular risk factors, such as total cholesterol, LDL, HDL, triglycerides, fasting glucose, SBP and DBP. In order to explore potential heterogeneity in estimates of treatment effect, we performed univariate meta-regression and compared summary results from subgroup analysis based on the type of anti-obesity drug, mean age, follow-up years, and study quality. We chose the random-effects model [19] with Mantel-Haenszel Statistics for all estimates of effect. Heterogeneity of treatment effects between studies was investigated visually by funnel plot and statistically by the heterogeneity I^2^ statistic. I^2^ statistic of 0%–40% indicates unimportant heterogeneity, 30%–60% indicates moderate heterogeneity, 50%–90% indicates substantial heterogeneity, and 75%–100% indicates considerable heterogeneity [20]. All reported P values were two-side and p values less than 0.05 were regarded as significant for all included studies. All analyses were carried out using STATA (version 10.0).

**Figure 3:**
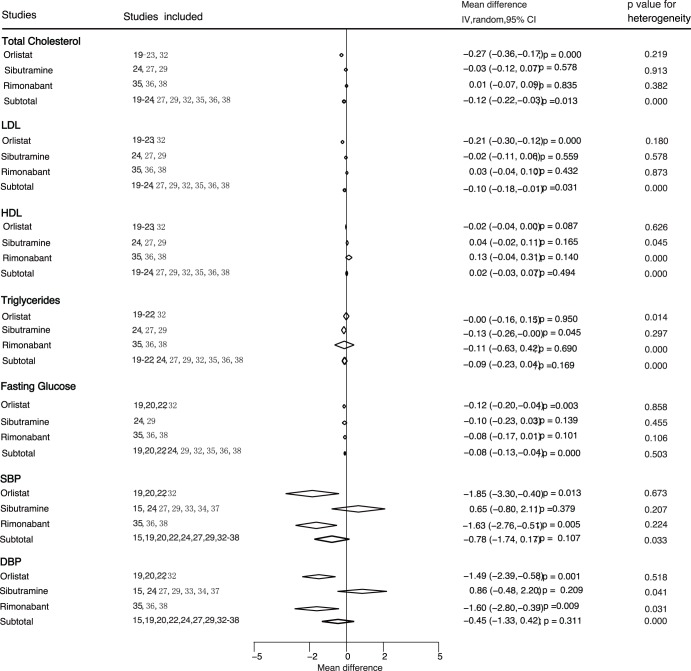
Subgroup analyses for the effects of anti-obesity on cardiovascular risk factors based on the type of drug. CI, confidence intervals; IV, inverse variance.

**Figure 4:**
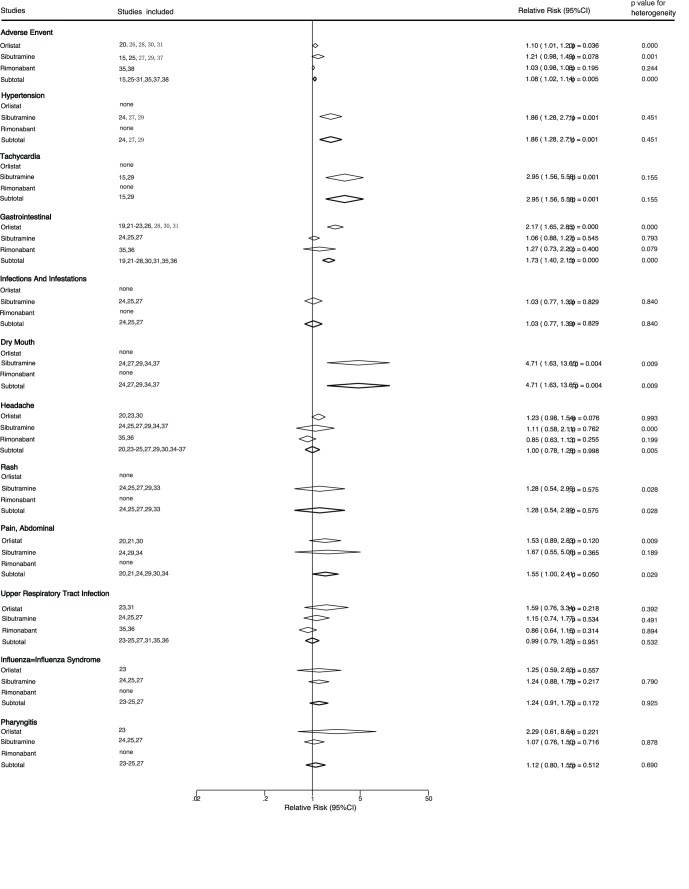
Summary of the relative risks of all adverse outcomes assessed.

## Results

We identified 957 potential articles from our initial electronic search, and 826 were excluded after a preliminary review of searches. The remaining 131 studies were retrieved for detailed assessment and 21 randomized controlled trials [15], [16], [21]–[39] met our inclusion criteria (Figure 1 and Protocol S1). The 21 trials provided data of 13759 people with obesity (mean: 655 people, range: 46 to 3277 people). Table 1 summarizes the baseline characteristics of the included studies and their participants. The follow-up for patients ranged from 4 to 48 months, with a mean of 14.7 months. Among these included trials, 10 studies evaluated orlistat therapy [21]–[25], [28], [30], [32]–[34], 3 [16], [37], [38] evaluated rimonabant therapy and remaining 8 [15], [26], [27], [29], [31], [35], [36], [39] evaluated sibutramine therapy. Because the included trials scarcely reported on the key indicators of trial quality, we assessed the quality of the trials by the pre-fixed criteria using the Jadad score. Overall, five trials scored 6, seven scored 5, six scored 4, two scored 3 and the remaining one scored 2.

**Table 2:** Subgroup analysis of weight loss, HDL and Triglycerides after treatment with anti-obesity agents.

		Subgroup		MD(95% CI)	P value	P value for heterogeneity
Weight loss	Orlistat	Mean age	>45	−1.17 [ −1.75, −0.58]	<0.001	0.86
			18–45	−3.06 [−3.92, −2.19]	<0.001	0.89
			<18	−	–	–
		Follow-up (month)	>12	−2.80 [−3.46, -2.14]	<0.001	0.44
			<12	−1.15 [−1.77, −0.53]	<0.001	–
		Jadad score	>4	−2.20 [−3.57, −0.84]	0.002	0.01
			<4	−2.66 [−3.86, −1.47]	<0.001	0.16
	Sibutramine	Mean age	>45	−0.10 [−0.78, 0.58]	0.77	–
			18–45	−3.89 [−4.92, −2.86]	<0.001	0.83
			<18	−5.37 [−8.20, −2.54]	<0.001	0.06
		Follow-up (month)	>12	−2.80 [−5.48, −0.11]	0.04	<0.01
			<12	−5.07 [−7.93, −2.21]	<0.001	0.02
		Jadad score	>5	−2.80 [−5.15, −0.45]	0.02	<0.01
			<5	−5.80 [−9.61, −1.99]	0.003	0.008
	Rimonabant	Mean age	>45	−3.66 [−4.17, −3.15]	<0.001	0.26
			18–45	–	–	–
			<18	–	–	–
		Follow-up (month)	>12	−3.66 [−4.17, −3.15]	<0.001	0.26
			<12	–	–	–
		Jadad score	>5	−3.67 [−4.55, −2.79]	<0.001	0.10
			<5	−3.60 [−4.36, −2.84]	<0.001	–
HDL	Orlistat	Mean age	>45	−0.04 [−0.23, 0.15]	0.69	–
			18–45	−0.02 [−0.04, 0.01]	0.16	0.37
			<18	–	–	–
		Follow-up (month)	>12	−0.02 [−0.04, 0.00]	0.09	0.63
			<12	–	–	–
		Jadad score	>4	−0.01 [−0.04, 0.01]	0.25	0.40
			<4	−0.05 [−0.11, 0.01]	0.09	0.83
	Sibutramine	Mean age	>45	0.00 [−0.04, 0.04]	1.00	–
			18–45	0.08 [0.00, 0.15]	0.04	0.20
			<18	–	–	–
		Follow-up (month)	>12	0.04 [−0.02, 0.11]	0.16	0.05
			<12	–	–	–
		Jadad score	>5	0.02 [−0.03, 0.07]	0.43	0.16
			<5	0.13 [0.02, 0.24]	0.02	–
	Rimonabant	Mean age	>45	0.13 [−0.04, 0.31]	0.14	<0.001
			18–45	–	–	–
			<18	–	–	–
		Follow-up (month)	>12	0.13 [−0.04, 0.31]	0.14	<0.001
			<12	–	–	–
		Jadad score	>5	3.61 [−3.47, 10.68]	0.32	<0.001
			<5	0.09 [0.06, 0.12]	<0.001	–
Triglycerides	Orlistat	Mean age	>45	−0.12 [−0.30, 0.06]	0.19	–
			18–45	0.08 [−0.20, 0.36]	0.58	0.006
			<18	–	–	–
		Follow-up (month)	>12	−0.00 [−0.16, 0.15]	0.95	0.01
			<12	–	–	–
		Jadad score	>4	−0.06 [−0.16, 0.05]	0.27	0.96
			<4	0.03 [−0.24, 0.31]	0.81	0.003
	Sibutramine	Mean age	>45	−0.06 [−0.19, 0.07]	0.38	–
			18–45	−0.24 [−0.43, −0.05]	0.01	0.72
			<18	–	–	–
		Follow-up (month)	>12	−0.13 [−0.26, −0.00]	0.05	0.30
			<12	–	–	–
		Jadad score	>5	−0.11 [−0.30, 0.08]	0.26	0.25
			<5	−0.22 [−0.44, −0.00]	0.05	–
	Rimonabant	Mean age	>45	−0.11 [−0.63, 0.42]	0.69	<0.001
			18–45	–	–	–
			<18	–	–	–
		Follow-up (month)	>12	−0.11 [−0.63, 0.42]	0.69	<0.001
			<12	–	–	–
		Jadad score	>5	5.45 [−5.89, 16.78]	0.35	<0.001
			<5	−0.41 [−0.56, −0.26]	<0.001	–

Data for the effect of anti-obesity drugs on weight loss compared with placebo were available from 16 trials (Figure 2). Overall, we noted that with anti-obesity therapy, weight was significantly reduced by 3.13 kg (95%CI: −4.00 to −2.26) compared with placebo. Furthermore, orlistat, sibutramine, and rimonabant produced reductions of 2.39 kg (95%CI: −3.34 to −1.45), 3.73 kg (95%CI: −6.00 to −1.46), and 3.66 kg (95%CI: −4.17 to −3.15) in weight, respectively.

Data for the effects of anti-obesity therapy on cardiovascular risk factors were divided into seven classes, i.e., total cholesterol, LDL, HDL, triglycerides, fasting glucose, SBP and DBP (Figure 3). Overall, orlistat therapy resulted in a reduction of 0.27 mmol/L (95%CI: −0.36 to −0.17) in total cholesterol, a reduction of 0.21 mmol/L (95%CI: −0.30 to −0.12) in LDL, a reduction of 0.12 mmol/L (95%CI: −0.20 to −0.04) in fasting glucose, a reduction of 1.85 mmHg (95%CI: −3.30 to −0.40) in SBP, and a reduction of 1.49 mmHg (95%CI: −2.39 to −0.58) in DBP. Moreover, sibutramine showed an effect on weight loss (MD = −3.73 kg, 95%CI: −6.00 to −1.46), triglycerides reduction (MD = −0.13 mmol/L, 95%CI: −0.26 to −0.00) with a statistical significance. Rimonabant showed clear effects on weight loss (MD = −3.66 kg, 95%CI: −4.17 to −3.15), SBP reduction (MD = −1.63 mmHg, 95%CI: −2.76 to −0.51), and DBP reduction (MD = −1.60 mmHg, 95%CI: −2.80 to −0.39).

We also noted that some adverse outcomes were reported in several trials and 12 trials reported data for total adverse event. Overall, anti-obesity therapy increased the risk of drug-related adverse events by 8% when compared with placebo (RR: 1.08, 95%CI: 1.02 to 1.14, Figure 4). Similarly, anti-obesity therapy also resulted in a increase of 73% in the risk of gastrointestinal diseases (RR: 1.73, 95%CI: 1.40 to 2.15), 86% in the risk of hypertension (RR: 1.86, 95%CI: 1.28 to 2.71), 195% in the risk of tachycardia (RR: 2.95, 95%CI: 1.56 to 5.58), and 371% in the risk of dry mouth (RR: 4.71, 95%CI: 1.63 to 13.65). Anti-obesity therapy did not have an effect on infections and infestations, headache, rash, abdominal pain, upper respiratory tract infection, influenza and pharyngitis.

We observed evidence of heterogeneity in the magnitude of the effect across the included trials for weight loss, HDL and Triglycerides. However, we performed a sequential exclusion of each trial from the pooled analysis and these exclusions did not affect our conclusions. Therefore, we did subgroup analyses to minimize the consequences of heterogeneity among the included trials based on mean age, follow-up years, and study quality (Table 2). Subgroup analyses were performed for weight loss, HDL and Triglycerides. The outcomes showed that there was evidence of heterogeneity in the magnitude of the effect across the included trials. The subgroup analysis based on the type of anti-obesity drugs contributed to the minimization of heterogeneity. However, few subsets provided a unanimous conclusion, and this might be due to the fact that fewer trials were included in these subsets.

## Discussion

Obesity remains the most frequent cause of cardiovascular disease, stroke, type 2 diabetes mellitus and other systemic diseases [40]. Evidence from several studies shows that obesity shortens life range from 4.8 to 7.1 years for people over forty years old, and it is associated with a tremendous social burden [41], [42]. Although many anti-obesity drugs show strong effects on weight loss, some researches [15], [43] indicate that the effects of anti-obesity drugs on the risk of cardiovascular risk factors remain unclear.

The results of this meta-analysis showed that anti-obesity therapy significantly reduced weight, total cholesterol, LDL and fasting glucose, and increased the risk of total adverse event, tachycardia, gastrointestinal diseases, hypertension, and dry mouth.

Due to the fact that the cardiovascular risk factors were increased in patients with diabetes mellitus, hyperlipidaemia, hyperglycemia, and hypertension [44], [45], we therefore restricted inclusion criteria to ensure our study to explore the effects of anti-obesity drugs on cardiovascular risk factors.

The participants in individual studies included in our meta-analysis could correspond with our eligible criteria. Patients with cardiovascular diseases or other cardiovascular risk factors should be excluded. Furthermore, the patients with uncontrolled hypertension, more than 4 kg weight loss in 3 months, surgery for weight reduction, a history of post surgical adhesions, bulimia or laxative abuse, use of any drug that might influence bodyweight or plasma lipids in the month before study entry, and drug or alcohol abuse did not meet our inclusion criteria either [15], [21]–[34].

Recently, several randomized controlled trials [41], [43], [46] indicated that anti-obesity therapy had direct effects on weight loss and cardiovascular risk factors in obese individuals. Furthermore, Daniels’s study [15] indicated that sibutramine might have some direct cardiovascular effects on obese adolescents, although they concluded that no evidence showed sibutramine produced an increase in the risk of cardiovascular risk factors, and the reason could be that cardiovascular risk factors might be affected by reduction in weight. Therefore, we restricted the baseline characteristics of obese individuals between these two groups to explore the correlation between anti-obesity drugs and cardiovascular risk factors.

Previous researches [45], [47] reported that anti-obesity drugs could reduce the body weight, contributing to an improvement in cardiovascular risk factors. However, the direct effect of anti-obesity drugs on cardiovascular risk factors is unclear. Furthermore, obesity usually accompanies with comorbidities, especially for older obese individuals. We therefore performed a systematic review and meta-analysis to assess intrinsic correlation between anti-obesity therapies and cardiovascular risk factors.

Previous meta-analysis [14] only demonstrated the effect of anti-obesity therapy on cardiovascular risk factors in adolescents, and provided weak correlation between them. However, this meta-analysis showed that anti-obesity therapy could reduce some cardiovascular risk factors, such as total cholesterol, LDL and fasting glucose, but had no effect on triglycerides, SBP and DBP.

Orlistat may play an important role in reducing SBP, DBP, total cholesterol, LDL and fasting glucose. The effect of orlistat on cardiovascular risk factors seems to be weak, although reduction in several cardiovascular risk factors was reported. However, the reason for this significant difference may arise from weight loss. Therefore, the reason for orlistat being superior to sibutramine could be that orlistat belongs to a class of anti-obesity agents that act directly and specifically at the site of fat breakdown in the lumen of the stomach and small intestine, so the effect of arlistat on cardiovascular risk factors seems to be weak [22].

Sibutramine produced an increase in several cardiovascular risk factors. Interpretation of this result is that receiving sibutramine includes the recognized effect of increased blood pressure, which increased the risk of cardiovascular events, especially for high risk people with previous cardiovascular disease [7]. Our research showed that hypertension in drug-related adverse event was also consistently higher in the sibutramine group than in the placebo group, which might have a direct effect on cardiovascular risk factors and contribute increase incidence of major cardiovascular events.

Rimonabant as a new drug, which is commonly used for obese people, produced a significant reduction in body weight. Moreover, it could effectively reduce SBP and DBP. Previous research [38] indicated that rimonabant therapy produced a substantial mobilization of abdominal fat, which would predict an improved cardiovascular risk profile. Rimonabant therapy alone will not eradicate the epidemic of obesity, however, our study provided evidence that rimonabant as a new approach could reduce the body weight and the cardiovascular risk.

Neither HDL nor Triglycerides was impacted by obesity medications, which might be due to the fact that the intrinsic effect might be lessened or balanced by other different anti-obesity drugs. For rimonabant therapy, it might produce an increase in HDL significantly, and the reason could be that only limited trials provided regarding data. For orslitat or sibutramine, it might have no direct effect on HDL or triglycerides.

The limitations of our research are shown as follows: (i) Inherent assumptions made by any meta-analysis–the analysis used pooled data either published or provided by individual study authors, and individual patient data or original data were unavailable, which restricted us from performing more detailed relevant analysis and obtaining more comprehensive results. (ii) The duration of follow-up and drug dose could affect our conclusions about the association between anti-obesity and cardiovascular risk factors. (iii) We did not have sufficient or appropriate data to explore detailed effects of anti-obesity therapy on body mass index, waist circumference and adverse events. (IV) Obese people frequently have low fat, low calorie, and AHA-type diet, which might play an important role in influencing cardiovascular risk factors.

Therefore, in future research, it is important to focus on patients with cardiovascular risk factors for primary prevention of cardiovascular disease, and to combine other anti-obesity drugs to provide an optimal therapy that minimizes adverse effects in obese individuals. We suggest that the ongoing trials should be improved in the following ways: (i) The adverse effects in clinical trials should be recorded and reported normatively, so that the side-effects of any treatment can be evaluated in future trials. (ii) The role of different drugs, dosages and treatment durations should be investigated in more detail to explore optimal anti-obesity drug, dose and duration of treatment [48]. (iii) Weight loss, body mass index and waist circumference should be recorded in detail to provide comprehensive information in the future trials.

## Supporting Information

Checklist S1 **PRISMA Checklist.**
(DOC)

Protocol S1 **PRISMA Flowchart.**
(DOC)
