# Supplementary material for: Effect of Anti-Obesity Drug on Cardiovascular Risk Factors: A Systematic Review and Meta-Analysis of Randomized Controlled Trials
Source: PLoS One. 2012 Jun 20;7(6):e39062. doi: 10.1371/journal.pone.0039062 (PMC3380040; doi:10.1371/journal.pone.0039062)
Supplement: Protocol S1 — PRISMA Flowchart. (DOC) [file pone.0039062.s002.doc]

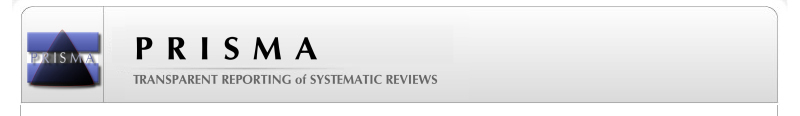
**PRISMA 2009 Flow Diagram**

**Screening**

**Included**

**Eligibility**

**Identification**

131 Full-text retrieve and review

110 Articles excluded

20 Not relevant

24 Reviews, letter to editor or not RCTs

28 The patients with other disease

16 The patients with other treatment

20 No desirable outcomes

2 Cross-over design

957 Potentially relevant trials identified

826 Articles excluded

339 Not relevant

219 Reviews, letter to editor or not RCTs

128 The patients with other disease

44 The patients with other treatment

42 Not English

45 No desirable outcomes

9 Cross-over design

21 Randomized controlled trials included in analysis
